# High-precision fabrication of 4m SiC aspheric mirror

**DOI:** 10.1038/s41377-022-01050-w

**Published:** 2023-01-01

**Authors:** Xiangang Luo

**Affiliations:** 1 State Key Laboratory of Optical Technologies on Nano-Fabrication and Micro-Engineering, Institute of Optics and Electronics, Chinese Academy of Sciences, 610209 Chengdu, China; 2 School of Optoelectronics, University of Chinese Academy of Sciences, 100049 Beijing, China

**Keywords:** Imaging and sensing, Astronomical optics

## Abstract

The 4 m diameter SiC aspheric mirror emerges due to a series of technological breakthroughs in blank mirror preparation, asphere fabrication, and testing, as well as cladding and coating, laying the groundwork for future research into large SiC mirrors for astronomical observation.

## Abstract (web summary)

Full process of large SiC mirror manufacture, promising to promote the next generation of engineering optics.

## Graphical abstract

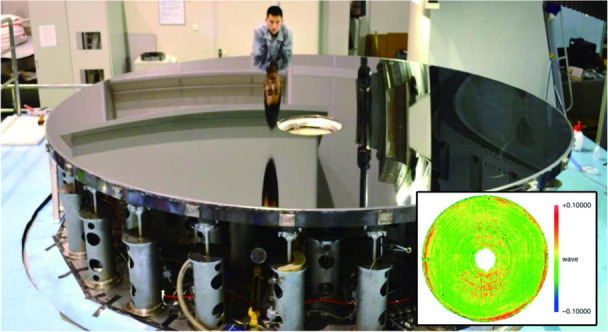

“Facing upwards to the blue sky, we behold the vast immensity of the universe; when bowing our heads towards the ground, we again satisfy ourselves with the diversity of species.” Human beings have shown great curiosity about the universe and ourselves since ancient times. Telescope is one of the most essential tools for universal research. Since a larger aperture benefits higher resolution and weaker signal detection, the size of telescopes has kept increasing since the first telescope was invented over 400 years ago. To achieve scientific goals for the coming decade, high-performance telescopes with a larger aperture and better wavefront error are urgently demanded^1,2^.

To build a large-size telescopic system, primary mirror manufacture^3^ is one essential work. Researchers pursue more free design and manufacture variables to improve imaging quality and reduce size and weight. Even though subwavelength optics^4^ and freeform optics^5^ show great potential in this aim, aspherical primary mirror was still the most common choice in recent systems. Meanwhile, to increase the size of mirrors and ensure high imaging quality in complex conditions, the areal density must be reduced while maintaining stiffness and stability^6^. Therefore, silicon carbide (SiC) has recently drawn significant attention in the telescope community worldwide because of its high specific stiffness and dimensional stability^7^.

However, the stringent requirements on material, surface type, full-spatial frequency (FSF) surface shape error, and surface roughness make manufacturing large SiC aspheric mirrors faces tough challenges due to the lack of systematic technology and equipment. Previously, the most giant SiC mirror was the one used as the primary mirror of the Herschel telescope, with a diameter of 3.5 m. The rough shape accuracy (3 μm) limited its working spectral range in far infrared and sub-millimeter^8^. In 2014, a large SiC mirror (i.e., 1.54 m × 0.49 m) available for the visible light field was reported by the French companies Reosc and Boostec^9^.

Recently, Xuejun Zhang and colleagues at Changchun Institute of Optics, Fine Mechanics and Physics, Chinese Academy of Sciences (CIOMP, CAS) have successfully manufactured a 4 m diameter SiC aspheric mirror, which is the largest reported worldwide (Fig. 1a). They summarized challenges in the fabrication process and their complementary strategies in the paper published on *Light: Science & Applications*^10^.

**Fig. 1:**
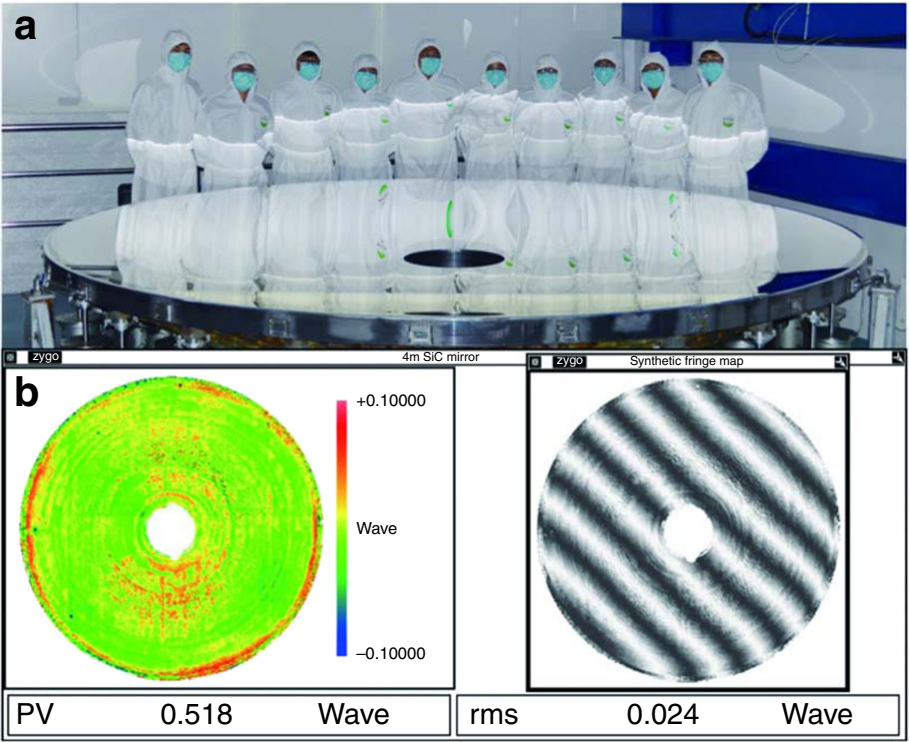
Four meter SiC aspheric mirror manufacturing results. **a** Picture of the 4 m SiC aspheric mirror. **b** Test result and interferogram of the ⌀4 m SiC aspheric mirror. Adapted with permission from ref. ^10^, Springer Nature

Several steps should be managed carefully in large-size mirror manufacturing, including blank preparation, lapping, polishing, cladding, etc. During the manufacturing process, accurate measurement across such a large area is essential as well. Xuejun Zhang and co-authors completed a comprehensive job among all these processes. To form lightweight SiC green bodies, water-soluble room temperature vanishing mold and gel casting technology was applied. Then the whole Φ4.03 m SiC blank was stitched by 12 segments of green bodies using vacuum reaction sintering^11^, and SiC powder was mixed in a binder to ensure the homogeneous joining^12^. Afterward, they developed an efficient converging algorithm based on frequency-domain correlation of surface error and a new optimized processing chain combining the CNC generating, stressed lap grinding/polishing, computer-controlled optical surfacing (CCOS), magnetorheological finishing (MRF) polishing and ion beam finishing. This combination enables high efficiency and accuracy fabrication with a 30% increase in error convergence rate. A low-temperature magnetron sputtering physical vapor deposition Si cladding process was proposed to replace the high-temperature chemical vapor deposition cladding process. The surface roughness of the Si cladding layer could be polished to 0.8 nm RMS. Meanwhile, to ensure the fabrication accuracy of the aspherical surface, a precision measurement toolset was developed, which seamlessly combines data from different test instrument. 6 nm RMS measurement accuracy and 2.6 nm RMS reproducibility were achieved, respectively. The errors caused by mirror self-gravity deflection and air turbulence^13^ were decoupled by gravity unloading and time-averaging techniques.

This aspheric mirror verified the large SiC mirror production capability. The aerial density of the SiC blank is less than 120 kg/m^2^, the thickness inhomogeneity of the cladding layer is less than 5%, and the final surface figure error and roughness are 15.2 nm RMS and 0.8 nm RMS, respectively (Fig. 1b). This mirror was delivered to customers in 2019, and applied in astronomical observation, earth exploration, and other areas. The key characteristics of this mirror ensure its great performance in those applications.

The high-precision manufacturing of a 4 m SiC mirror has overcome the difficulties of large-aperture lightweight silicon carbide material preparation technology, aspheric processing, modified coating technology, and measurement technology. We can expect that the performance of the proposed technique could be further promoted, pushing its application to a higher level. This technique, along with the rapid developments of large-size and lightweight planar optical elements^4^ and mirror segmented strategies^14,15^, is promising to promote the next generation of engineering optics.
